# Development of Hand Therapy as a specialty

**DOI:** 10.1186/1753-6561-9-S3-A108

**Published:** 2015-05-19

**Authors:** Sarah Ewald

**Affiliations:** 1 City Handtherapie, Zurich, 8006, Switzerland

## 

In some areas of the world Hand Therapy as a specialty practice area is well established, where as in other areas of the world it is just developing. The International Federation of Societies for Hand Therapy (IFSHT), founded in 1989, supports the development of Hand Therapy worldwide. IFSHT’s mission is: to provide global networking & educational opportunities to develop & enhance the practice of hand therapy worldwide. IFSHT has 44 member countries. In 33 member countries Hand Therapy societies have been established. In 9 member countries, an individual therapist has been accepted as a corresponding member of IFSHT as a Hand Therapy society does not yet exist in that country. IFSHT welcomes corresponding members for a limited time period, with the aim of supporting the development of a Hand Therapy interest group or Hand Therapy society. With the establishment of a dedicated Hand Therapy society in a country, like minded therapists are able to come together and network with one another to further the development and establishment of Hand Therapy in their country.

In July and August 2014, IFSHT surveyed its full membercountries with the aim of identifying trends in Hand Therapy development. An e-survey with 22 questions about the practice of Hand Therapy was sent (in English) by e-mail to the 33 IFSHT national delegates. A total of four reminders were sent in addition to the initial invitation. A response rate of 87.8% was attained. Twenty nine of 33 countries completed the survey. Information from the ongoing needs survey of 9 corresponding members was included as where pertinent. Data from IFSHT membership records was utilized for the four countries (Finland, Korea, Spain and Venezuela) that did not complete the survey. Data on country population was obtained on July 31, 2014 from www. countrywatch.com.

**Number of Hand Therapists worldwide:** In total there are 8385 Hand Therapists who are members of IFSHT. Approximately, 70% are Occupational Therapists (OT) and 30% are Physical Therapists (PT) who have specialized in Hand Therapy. The proportion of OT’s and PT’s who specialize in Hand Therapy varies from country to country. The most extreme examples of this are Turkey where only PT’s specialize and Denmark where 99% of Hand Therapists are OT’s.

**Density of Hand Therapy professionals to population:** Among IFSHT members, eleven countries have less than 100,000 people served by 1 Hand Therapist. The greatest density of Hand Therapists per capita is in Finland, where there is reported to be one Hand Therapist per 17, 218 people. Fifteen member countries have between 100,001 and 999,999 people served by one Hand Therapist. Sixteen countries have 1,000,000 or more people for each Hand Therapist.

**Educational opportunities for Hand Therapists:** Respondents indicated that participation Hand Therapy skills courses was the most frequently utilized method for therapists in their country to learn about Hand Therapy. Other opportunities that were often utilized included visiting programs, hand therapy internship programs and post-graduate certificate programs.

**Accreditation for Hand Therapists:** Thirteen countries have recognition programs in place for therapists that specialize in Hand Therapy. Three types of recognition programs were identified: **formal educational programs** leading to a diploma or certificate in Hand Therapy, **portfolio programs** that recognize experience and require evidence of knowledge, **auto-didactic programs** that issue certification upon completion of a Hand Therapy exam. The renewal requirements for accreditation vary by country. The majority of countries have set the renewal period at five years.

**Access to Hand Therapy information:** Respondents were asked aboutthe availability of Hand Therapy literature in their country in their language. Seven countries reported that there are no Hand Therapy books are available in comparison eight countries reported that there were more than 10 Hand Therapy books available. In 12 countries a Hand Therapy journal is available and in 14 countries no such journal is available. In the age of the internet, access to information is easier than ever. However respondents indicated that language and the expense of acquiring information are barriers to acquiring information relevant to Hand Therapy in their country.

**Facilitating the development of Hand Therapy:** Eighty-six percent of respondents indicated that their national society supported the development of Hand Therapy by: organizing national Hand Therapy Congresses, having a Hand Therapy society website, and by organizing Hand Therapy courses.

IFSHT strives to support the development of Hand Therapy worldwide by connecting therapists with one another. The IFSHT website (www.ifsht.org) offers information: about IFSHT, the practice of Hand Therapy, lists clinics that are open to hosting visitors, lists organizations that offer hand therapy volunteer opportunities, lists Hand Therapy relevant websites and much more. IFSHT also contributes articles about Hand Therapy to the IFSSH E-zine, which is an open-access publication. The IFSHT Update contains information about Hand Therapy and upcoming meetings worldwide and is published four times a year in Hand Therapy journals as well as on the IFSHT website. IFSHT also offers a Hand Therapy connections e-newsletter, which may be subscribed to via the IFSHT website. Every three years an IFSHT congress is hosted in a different location. The next IFSHT congress, with a scientific and social program will be in Buenos Aires, Argentina, October 24-28, 2016. More information is available at http://www.ifssh-ifsht2016.com.

